# Seasonality and weather conditions jointly drive flight activity patterns of aquatic and terrestrial chironomids

**DOI:** 10.1186/s12898-018-0175-y

**Published:** 2018-06-19

**Authors:** Lucie Vebrová, Andre van Nieuwenhuijzen, Vojtěch Kolář, David S. Boukal

**Affiliations:** 1 Department of Ecosystem Biology, Faculty of Science, University of South Bohemia, Branišovská 31a, 37005 Ceske Budejovice, Czech Republic; 2 Freelance Researcher, Roseč, Czech Republic; 3 Institute of Entomology, Biology Centre, Czech Academy of Sciences, 37005 Ceske Budejovice, Czech Republic

**Keywords:** Aquatic insects, Dispersal, Seasonality, Environmental conditions, Collinearity

## Abstract

**Background:**

Chironomids, a major invertebrate taxon in many standing freshwaters, rely on adult flight to reach new suitable sites, yet the impact of weather conditions on their flight activity is little understood. We investigated diel and seasonal flight activity patterns of aquatic and terrestrial chironomids in a reclaimed sandpit area and analysed how weather conditions and seasonality influenced their total abundance and species composition.

**Results:**

Air temperature, relative humidity, wind speed, and air pressure significantly affected total flight activity of both groups, but not in the same way. We identified an intermediate temperature and humidity optimum for the flight activity of terrestrial chironomids, which contrasted with weaker, timescale-dependent relationships in aquatic species. Flight activity of both groups further declined with wind speed and increased with air pressure. Observed flight patterns also varied in time on both daily and seasonal scale. Flight activity of both groups peaked in the evenings after accounting for weather conditions but, surprisingly, aquatic and terrestrial chironomids used partly alternating time windows for dispersal during the season. This may be driven by different seasonal trends of key environmental variables in larval habitats and hence implies that species phenologies and conditions experienced by chironomid larvae (and probably other aquatic insects with short-lived adults) influence adult flight patterns more than weather conditions.

**Conclusions:**

Our results provide detailed insights into the drivers of chironomid flight activity and highlight the methodological challenges arising from the inherent collinearity of weather characteristics and their diurnal and seasonal cycles.

**Electronic supplementary material:**

The online version of this article (10.1186/s12898-018-0175-y) contains supplementary material, which is available to authorized users.

## Background

Long-term survival of species in changing environments depends on the ability of local populations to reach new suitable sites [1, 2]. This is particularly true for biota in highly dynamic, small standing freshwaters [3]. Dominant invertebrate groups occupying these habitats utilize different strategies for dispersal, defined as any movement between spatially or temporally discrete localities or populations [2]. While zooplankton disperses mostly as resting eggs, nearly all aquatic insects disperse between discrete localities by flight as adults [3]. Their seasonal flight patterns reflect species phenologies and long-term environmental conditions affecting the pre-adult stages.

Various biotic and abiotic factors, including weather conditions, provide environmental filters that modify adult flight patterns on daily timescales [4]. These constraints ultimately shape dispersal patterns of aquatic insects at diel and seasonal timescales [5–7] that may affect community assembly through priority effects [8] and metapopulation dynamics [9]. However, teasing apart the contribution of species phenology and environmental conditions to flight patterns is inherently difficult due to ubiquitous strong collinearity of environmental variables and their seasonality in many ecosystems [10].

Flight activity of aquatic insects is strongly affected by weather conditions including air temperature [6, 11, 12], wind speed [12–14], light intensity [15, 16], and relative air humidity (i.e., the amount of water vapour in the air relative to the full saturation) [17, 18]. Weather conditions thus represent a strong environmental filter [19, 20] that ultimately affects individual fitness. Temperature can drive species composition of the colonizing insects because few individuals fly at temperatures outside the species-specific thermal windows of flight activity [16]. Relative air humidity provides an important constraint as flight during inappropriate conditions may increase the risk of dehydration and mortality as lower relative humidity leads to faster drying [5]. Joint impact of air temperature and relative humidity on flight activity of aquatic insects was recognized for many groups [21–23] including chironomids [24], but the individual contributions of humidity and temperature are often difficult to separate due to their collinearity [10]. Dispersal flight of aquatic beetles and heteropteran bugs is further inhibited by wind [13, 14] and modified by light intensity and solar elevation, which determines the amount of horizontally polarized light reflected by water surface that helps individuals locate suitable habitats and oviposition sites [25].

To our knowledge, the joint effects of weather conditions and seasonality on flight activity in aquatic insects were studied only in stream Plecoptera [12] and *Culicoides* biting midges [23] but are virtually unknown in chironomids, a highly speciose and abundant macroinvertebrate group in fresh waters [26]. Chironomids are generally poor fliers that disperse by wind [27] rather than through self-propelled flight within their ‘flight boundary layer’ (sensu [28]). The adults are mostly short lived [29] and their presence is driven by larval phenology, while weather conditions strongly affect their flight activity that should primarily relate to reproduction and oviposition, although adults may also seek plant food sources such as nectar, pollen and honeydew [27, 30, 31].

Small and soft-bodied insects such as chironomids are also more vulnerable to weather conditions because they can cool down, overheat or desiccate rapidly [10] and stop flying in strong winds [32]. These differences in key individual characteristics related to dispersal suggest that environmental conditions may affect chironomids differently from other aquatic insect groups, with potential implications for the process of colonization of new habitats and local community assembly [33]. Moreover, chironomids include both aquatic and terrestrial species [29] and understanding differences in their flight patterns (if any) could shed more light on environmental drivers of dispersal activity of aquatic insects. Chironomids living in standing waters can move between different microhabitats within the same water body as first-instar larvae [27], but cannot cross land in the larval stage. Adult flight is therefore crucial for their dispersal between water bodies, while terrestrial species in more contiguous habitats might in principle combine dispersal in both larval and adult stage. Previous comparisons of aquatic species (i.e., with larvae living in various aquatic habitats) and terrestrial species (i.e., with larvae living in wet soil or damp vegetation [27]) of chironomids focused only on differences in spatial distribution [34, 35].

The main purpose of this paper was to investigate and compare the diel and seasonal flight activity patterns of aquatic and terrestrial chironomids, focusing on their assemblage in a reclaimed sandpit as a case study. We characterized the patterns and disentangled the effects of temporal and environmental factors on flight activity of both groups. We expected a strong dependence of flight activity on wind speed, temperature and relative humidity because chironomids rely on dispersal by wind and have relatively soft bodies, but had no a priori expectation on the differences between flight patterns of aquatic and terrestrial species.

## Methods

### Study site

The study was carried out in the Cep II sandpit near Suchdol nad Lužnicí, Czech Republic (GPS 48°91′85.51′′N, 14°87′42.58′′E). Most of the sandpit area is covered by a deep and turbid lake with ongoing sand extraction [36]. Shore area in the south-western part of the sandpit was remodelled and a cluster of ca. 40 small temporary to permanent pools (clayey-sandy bottom, surface area: mean ± SD = 8.6 ± 3.5 m^2^; depth, 0.28 ± 0.09 m) created there in October 2012 to conduct a community assembly experiment. Aquatic invertebrates began to colonize the pools in early 2013 (Boukal et al. unpublished data). Immediate vicinity of the pools consisted of bare clayey-sandy ground with very sparse cover of herbs and no shrubs. We sampled flying chironomids near the pools (Additional file 1: Figure S1) using two standard methods: sweeping with a hand-held aerial net and Malaise traps.

### Diel flight activity in autumn 2013

To observe the diurnal flight patterns of chironomids and select the method for the subsequent long-term study, we sampled flying insects with a handnet and Malaise traps for 8 days in late summer (11–15 August and 23–25 September 2013). Sweeping was carried out with a handnet with 55 cm diameter and a white mesh. One of us (LV) continuously swept the air at ~ 1 m height while walking slowly (ca. 4 km h^−1^) for 15 min along one of two predetermined, equally long routes, one closer to the lake shore and another closer to the largest pool (Additional file 1: Figure S1). This routine was performed 11 times every hour from 9:00 to 19:00 CET (Additional file 1: Table S1) with regular alternation between the two routes each day and between days. Insects were removed from the handnet with an aspirator and preserved in 80% ethanol.

Four Malaise traps (Additional file 1: Figure S2) were deployed on the same dates, two near the lake shore and two near the largest pool (distance within each pair: ca. 12 m, minimum distance between pairs: ca. 30 m). Traps within each pair were placed perpendicular to each other to minimize potential biases caused by wind direction. Malaise traps are generally considered non-attractive for flying insects, although we cannot rule out the possibility that some species might use them as landmarks towards which they direct their swarming activity. Intercepted flying insects were accumulated in a 0.5-L bottle attached to the top of the trap and filled with glycerated 80% ethanol. Traps were exposed continuously between 8:00 and 20:00 CET and the entire sample was collected afterwards.

Local air temperature and humidity were monitored every 15 min with two data loggers (Ebro EBI 20-TH) placed 1 m above the ground in a shaded ventilated space, one near the lake and another near the pools. We also recorded cloud cover (four categories: clear sky, mostly sunny, mostly cloudy, and cloudy) and wind speed on the Beaufort scale (Additional file 2: Table S2). Beaufort scale was used directly in statistical analyses of the 2013 data and then converted to m s^−1^ using a regression between the wind speeds recorded on the Beaufort scale, and in m s^−1^ for the 2014–2015 data to facilitate the comparison between the results on diel and seasonal flight activity.

### Seasonal flight activity in 2014–2015

Following a comparison of both sampling methods for the 2013 data (see “Results”), we deployed the same four Malaise traps for four consecutive days every month between May and Sept. 2014 and in Mar. and Apr. 2015 to study the seasonal flight patterns. The traps were placed as in 2013 but the samples were collected 5 times a day every 4 h (first at 6:30 and the last at 22:30 CET). Based on the results from 2013 along with preliminary inspection of the 2014–2015 samples, we analysed only data on chironomids captured in the afternoon and evening covering the sunset (from 14:30 to 22:30 CET). We pooled data from each trap on each day as one sample and used only average values of environmental characteristics during this period (Additional file 1: Table S1). Our results thus convey the daily average response of chironomids, but the weather on the sampling dates was relatively stable and the average environmental characteristics were strongly collinear with their minima and maxima (Additional file 2: Table S3).

Air temperature and humidity on the site were recorded as in 2013. In addition, hourly data on average air pressure and wind speed and point data on cloud cover (0–10 scale; recorded at 7:00, 14:00, and 22:00; Additional file 2: Table S2) were obtained from the field site of the Czech Hydrometeorological Institute in Třeboň, 12 km away from the study site. We used air temperature and relative humidity data measured at our experimental site; they were close to the data from Třeboň (Additional file 2: Figure S4). No rain occurred on the sampling dates.

Adult males were identified under Olympus SZX9 and Olympus BH microscopes using keys and descriptions [37–40] to the species or genus level except most terrestrial Orthocladiinae from 2013, which were grouped together. Females were excluded from the analyses because their identification except a few species is difficult or impossible [41].

### Statistical analysis

We carried out five analyses: (1) calculation of species rarefaction curves [42] to compare the two sampling techniques used in 2013, (2) univariate analysis of time- and weather-dependent changes in total abundance of adult chironomids in 2013 and in 2014–2015, (3) multivariate analysis of seasonal and weather-dependent changes in the composition of chironomid assemblages in 2014–2015 including variation partitioning to detect pure effect of season and environmental factors, (4) analysis of seasonal flight phenology of common species in 2014–2015 using species response curves, and (5) multivariate analysis of seasonal flight patterns in 2014–2015 including the larval habitat as species trait. In order to detect possible differences in flight patterns explained by larval habitat, we performed the second, third and fourth analysis separately for aquatic and terrestrial species; the latter also included the few rare semi-terrestrial species. Univariate analyses and calculations of rarefaction curves were done in R version 3.1.2 [43]. Multivariate analyses and species response curves were calculated in CANOCO 5 [44].

Rarefaction analysis was implemented in the *iNEXT* package version 2.0.8 [45] with the number of individuals as the rarefaction unit. Samples from each part of the day and each method were aggregated regardless of locality, but we also ran a supplementary analysis with data split by locality. We calculated rarefaction curves for the whole chironomid assemblage and for aquatic species only. The results were used to select the sampling method for the survey of seasonal patterns in 2014–2015.

Generalized linear models (GLMs) were used to analyse the effects of air temperature *T*, relative humidity *H*, wind speed *W*, cloud cover *C*, air pressure *P* (the latter only in 2014–2015) and location *loc* on the total abundance of chironomids in the handnet samples in 2013 and in the Malaise trap samples in 2014–2015. We used the 2013 handnet samples as they had higher temporal resolution than the Malaise trap samples. We ignored spatio-temporal autocorrelations in the models because adult chironomids are short-lived and relatively poor fliers. We thus assumed that (i) sampling did not remove individuals that could be found later, i.e., individuals caught by the Malaise trap on a given day would not be present at the site next day, individuals caught by the handnet were flying away or inside the experimental site and would not be caught during the next sampling after 1 h, and (ii) distances between the traps and handnet sampling routes were sufficient to ensure independent samples.

All five environmental variables (*T*, *H*, *C*, *P*, and *W*) were standardized and the resulting z-scores included as second-order orthogonal polynomials to model nonlinear responses except a few cases explained below. To detect possible inter-correlation among weather parameters, we computed Pearson correlation coefficients (Additional file 2: Table S4). As expected, air temperature and relative humidity were strongly collinear, and we thus used them as explanatory variables separately for both datasets. We also detected two distinct weather regimes with highly collinear temperature and humidity (one in May–Sept. 2014 and another in Mar. and Apr. 2015; Additional file 2: Figure S4d). We thus considered a weather regime (categorical variable *TH*, set to 0 in 2014 and to 1 in 2015) in addition to humidity or temperature in some models. We included the respective effect of daytime (*time*, continuous) and season (either as continuous *season* running from 1 Jan. to 31 Dec. and scaled linearly between − 0.5 and 0.5, or as discrete *month*) for the 2013 and 2014–2015 data.

We created respectively two and four saturated models D1–D2 and S1–S4 of diel and seasonal flight patterns and applied each of them separately to aquatic and terrestrial species. For diel flight patterns, we evaluated the effect of location, month and current weather conditions on the total abundance *N* of adults captured in the handnet. Nonlinear responses were considered except cloud cover, which was treated as a factor (*C*_*F*_) in 2013:

D1 $$N\sim month{\kern 1pt} \, + \,Q(time) + Q(T)\, + C_{F} + Q(W) + loc$$

D2 $$N\sim month\, + \,Q(time) + Q(H)\, + C_{F} + Q(W) + loc$$

where *Q*(*x*) stands for a second-order orthogonal polynomial to detect a nonlinear response to the variable *x.* Using the same approach, we compared four models of seasonal flight activity:

S1 $$N\sim month\, + \,Q(T) + Q(C)\, + Q(W) + Q(P) + loc$$

S2 $$N\sim month{\kern 1pt} \, + \,Q(H) + Q(C)\, + Q(W) + Q(P) + loc$$

S3 $$N\sim season{\kern 1pt} \, + \,Q(T) + TH + Q(C)\, + Q(W) + Q(P) + loc$$

S4 $$N\sim season{\kern 1pt} \, + \,Q(H) + TH + Q(C)\, + Q(W) + Q(P) + loc{\kern 1pt}$$

Data were overdispersed and we thus used quasi-Poisson distribution. For each of the full models D1–D2 and S1–S4, we performed manual stepwise selection based on quasi-AIC_c_ criterion corrected for small sample size (qAIC_c_; [46]) with repeatedly extracted overdispersion parameter to select the final model. To identify the overall best model describing the daily and seasonal pattern, we compared the resulting final models based respectively on D1–D2 and on S1–S4 using qAIC_c_ with the dispersion parameter calculated from a new saturated model containing all explanatory variables included in D1–D2 and in S1–S4 (*season* and *TH* were left out from the saturated model based on S1–S4 because they were determined by *month*). This step was required as the competing final models derived from D1–D2 or S1–S4 were not necessarily nested; using other plausible values of the dispersion parameter, e.g., from one of the competing models, lead to qualitatively same conclusions (results not shown).

We calculated McFadden’s pseudo-R^2^ for each final model based on D1–D2 and S1–S4 as the difference between null and residual deviance divided by null deviance of the model [47]. We observed no highly influential observations, the residuals of final models were approximately homoscedastic and showed no clear trends against the explanatory variables, and adding a quadratic dependence on season did not improve the fit of models S3 and S4 for both terrestrial and aquatic species. Significance of all explanatory variables in the final models was assessed by a likelihood ratio test and model fits illustrated using the *effects* package version 3.0–6 [48].

We further assessed seasonal flight patterns and the effect of environmental parameters on species composition of chironomid assemblages using redundancy analysis (RDA) for the 2014–2015 Malaise trap data. Species abundances (*n*) were transformed as log_10_(*n *+ 1) and centred before analyses. Single-term ordinations were separately computed for the season and environmental parameters. Pure effect of each weather characteristics was identified using partial RDA with season as a covariate. We also computed variation explained only by season, only by environmental factors and shared variation by variation partitioning. In order to explain any differences between flight activity of aquatic and terrestrial chironomid species, we used the same RDA analysis as above on the pooled aquatic and terrestrial species data with larval habitat as a species trait. To minimize the influence of rare or randomly recorded species in all multivariate analyses, we used species with at least 5 occurrences in the data for both aquatic and terrestrial species.

Seasonal changes in flight activity of the most frequently found species were illustrated by species response curves implemented as generalized additive models (GAMs) with quasi-Poisson distribution. We started with five degrees of freedom that were reduced for each species based on stepwise selection using AIC. We used total abundance pooled across all four traps per day as the response variable and month as the time variable for 2014–2015 data (Additional file 1: Table S1). All models were based on Monte Carlo tests with 9999 unrestricted random permutations.

## Results

The local chironomid assemblage was highly diverse and included 31 aquatic, five terrestrial, one semi-terrestrial species, and two species with unknown habitat association in August and September 2013 (*n* = 2467 males). Malaise trap samples in 2014–2015 represented 65 aquatic, eight terrestrial, and two species with unknown habitat association (*n* = 2356 males). These numbers might slightly underestimate the true species richness because a small proportion of the taxa could not be fully identified and might have included multiple species (Additional file 3: Table S5). Species composition differed between years, with only 27% species collected in August and September shared between both datasets; these species were common throughout the study. Abundances were highly skewed; only three and four identified species were common (> 100 males) in 2013 and 2014–2015, respectively.

**Fig. 1:**
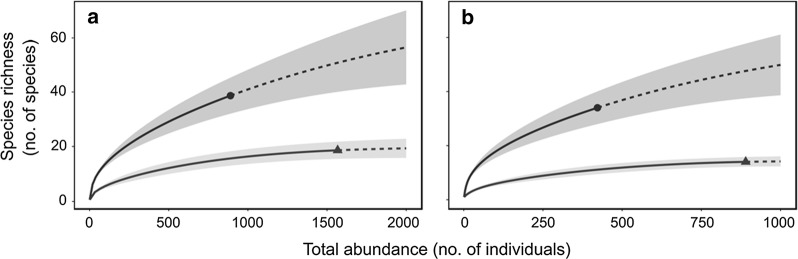
Rarefaction curves (data: solid lines, extrapolation: dashed lines) of the net (line with triangle), Malaise trap (line with dot) for **a** whole chironomid assemblage and **b** only for aquatic species. Shaded areas indicate 95% confidence intervals

Handnet and Malaise traps differed significantly in their ability to cover the 2013 chironomid assemblage (Fig. 1 and Additional file 4: Figure S5). Although we caught more males with the handnet (*n *= 1567) than with the Malaise traps (*n *= 890), the latter yielded more taxa (handnet: 20; Malaise traps: 41) and suggested a much more speciose local assemblage (mean species diversity at 3000 individuals predicted by rarefaction, with 95% CI in parentheses: handnet, 21.2 (16.8–25.7) species; Malaise traps, 68.8 (46.7–90.8) species; Fig. 1). Differences between both sampling sites were small, especially for the Malaise traps (Additional file 4: Figure S5). We thus chose Malaise traps to survey seasonal flight patterns in 2014–2015.

### Diel flight activity in autumn 2013

Flight activity of both terrestrial and aquatic chironomids changed in time and with weather conditions. Final models for both groups favoured temperature (D1) over humidity (D2) as the main driver of their total flight activity, although both model types were plausible (∆qAIC_c_ ≤ 1.7). All four final models explained a large proportion of variability in the data, especially for the aquatic taxa (Table 1).

**Table 1:** Summary of the final models of the total diel and seasonal flight activity of adult chironomids

Model	∆qAICc	d.f.	*w*	R^2^
Diel pattern: aquatic species, 2013 data				
D1: *N* ~ *month *+* Q*(*time*)+ *T *+* loc*	0.0	9	0.70	0.77
D2: *N* ~ *Q*(*time*)+* H *+* W*^*2*^+* loc*	1.7	6	0.30	0.73
Diel pattern: terrestrial species, 2013 data				
D1: *N* ~ *month* + *time*^*2*^+ *T*^*2*^+* W*^*2*^	0.0	5	0.54	0.55
D2: *N* ~ *month *+* Q*(*H*) + *W*^*2*^	0.3	5	0.46	0.54
Seasonal patterns: aquatic species, 2014–2015 data				
S2: *N* ~ *month* + *H *+ *W *+* P *+* loc*	0.0	11	0.58	0.71
S1: *N* ~ *month* + *T *+* W *+* P *+* loc*	1.6	11	0.25	0.70
S3: *N* ~ *season* + *Q*(*T*)+ *TH *+* W *+* P *+* loc*	2.5	8	0.17	0.68
S4: *N* ~ *season* + *Q*(*H*)+ *C *+* W *+* P *+* loc*	13.9	8	< 0.001	0.64
Seasonal patterns: terrestrial species, 2014–2015 data				
S1: *N* ~ *month* + *T*^*2*^+ *W *+* P *+* loc*	0.0	11	0.76	0.64
S2: *N* ~ *month* + *H*^*2*^+ *C *+ *W *+* P* +* loc*	2.3	12	0.24	0.65
S3: *N* ~ *Q*(*T*)+ *TH *+* C *+* Q*(*W*)	48.6	7	< 0.001	0.44
S4: *N* ~ *H* + *Q*(*W*)	63.6	4	< 0.001	0.36

Corresponding initial saturated model given in front of each final model. ∆qAIC_c_, difference in qAIC_c_ from the model with the lowest qAICc value; d.f., degrees of freedom; *w*, qAIC_c_ weight; R^2^, McFadden’s pseudo-R^2^. See “Methods” for abbreviations of variables and saturated models; *H*^*2*^, *T*^*2*^ and *W*^*2*^ = linear term not retained in the final model; *Q*(*x*) = a second-order orthogonal polynomial of the variable *x*. Terrestrial species also include semi-terrestrial taxa

**Fig. 2:**
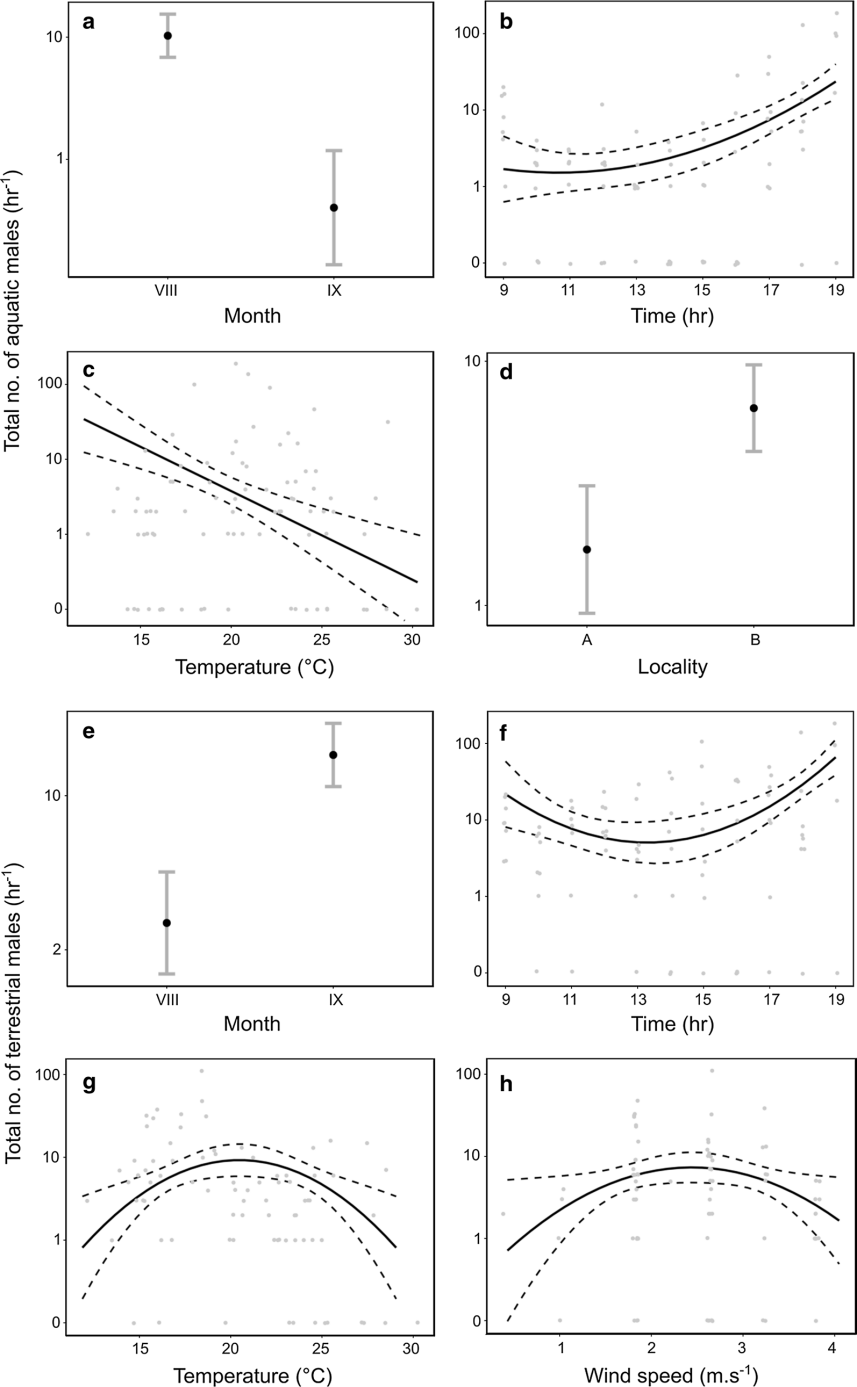
Drivers of diel patterns of total flight activity of **a**–**d** aquatic and **e**–**h** terrestrial species. Locations: A = close to lake shore and B = close to experimental pools. Solid black lines and black points = model fit; dashed lines and grey error bars = 95% confidence intervals; grey points = overlaid raw data with small amount of jitter added. Y-axis on log_10_ scale with zeroes placed at 0.1

Temporal and environmental drivers of total flight activity were similar but not identical across the models and groups. Flight activity of both aquatic and terrestrial chironomids peaked in the evening, with a second lower peak in the morning, and this time dependence was retained in three of the four final models (aquatic, model D1: F_2, 75_ = 60.5, *P* < 10^−4^; aquatic, D2: *F*_2, 78_ = 71.8, *P* < 10^−4^; terrestrial, D1: F_2, 73_ = 8.4, *P* = 0.004; Fig. 2 and Additional file 4: Figure S6). Flight activity of aquatic species further decreased with temperature (D1, linear term: F_1, 75_ = 26.5, *P* < 10^−4^) or relative humidity (D2, linear term: F_1, 78_ = 20.7, *P* < 10^−4^) and the distance from experimental pools (D1: F_1, 75_ = 32.4, *P* < 10^−4^; D2: F_1, 78_ = 19.4, *P* < 10^−4^), and was marginally affected by wind (D2: F_2, 78_ = 2.4, *P* = 0.08). This contrasted with a unimodal dependence of flight activity of terrestrial species on air temperature (D1: F_2, 73_ = 9.2, *P* = 0.003) or relative humidity (D2: F_2, 75_ = 14.0, *P* < 10^−4^) and wind speed (D1: F_2, 73_ = 7.2, *P* = 0.009; D2: F_2, 75_ = 3.4, *P* = 0.03) with the respective estimated maxima of flight activity near 20 °C, 40% relative humidity, and wind speeds of 2.5 m s^−1^. We found no significant effect of cloud cover on either group. The patterns in three of the four models were confounded by differences between months, with aquatic species flying predominantly in August and terrestrial ones in September (aquatic, D1: F_1, 75_ = 41.0, *P* < 10^−4^; terrestrial, D1: F_1, 73_ = 50.8, *P* < 10^−4^; terrestrial, D2: F_1, 75_ = 45.1, *P* < 10^−4^).

### Seasonal flight activity in 2014–2015

Flight activity of both terrestrial and aquatic chironomids also varied markedly during the season. We recorded the highest flight activity in June 2014 (32% of all males), July 2014 (20%), and April 2015 (19%), which contrasted with very few individuals collected in March. 2015 (0.2%). Environmental gradients were strong (Additional file 2: Table S2) and included extreme values beyond which flight activity completely ceased. For example, the minimum average afternoon and evening temperature at which any adults were caught was 5.2 °C on 15 March 2015, well above the minimum average recorded temperature (− 1.7 °C).

**Fig. 3:**
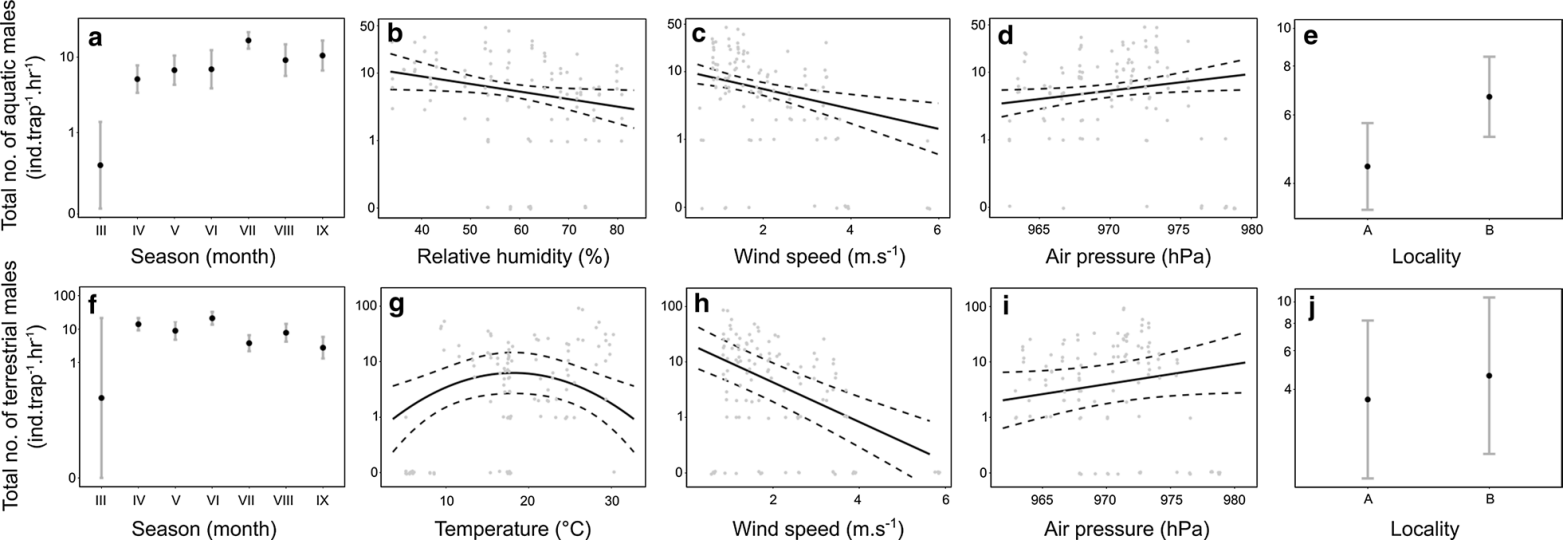
Drivers of seasonal flight activity of total flight activity of **a**–**e** aquatic and **f**–**j** terrestrial chironomids. Symbols and axes as in Fig. 2

Seasonal flight activity patterns were similar but not identical in aquatic and terrestrial chironomids (Table 1 and Fig. 3). Plausible models (∆qAIC_c_ ≤ 2, aquatic: S1 and S2; terrestrial: S1) showed that the flight activity of both groups varied strongly between months (aquatic, S2: F_6, 101_= 20.1, *P* < 10^−4^; aquatic, S1: F_6, 101_ = 10.0, *P* < 10^−4^; terrestrial, S1: F_6, 100_ = 19.8, *P* < 10^−4^). Estimated highest flight activity of aquatic adults under mean weather conditions (i.e., with weather conditions averaged across the whole dataset) fell in July followed by September, which contrasts with the estimated maxima in April and June for terrestrial taxa (Fig. 3).

Total flight activity of both groups increased with air pressure (aquatic, S2: F_1, 101_ = 5.2, *P* = 0.02; aquatic, S1: F_1, 101_ = 6.5, *P* = 0.01; terrestrial, S1: F_1, 100_ = 3.6, *P* = 0.06), decreased with wind speed (aquatic, S2: F_1, 101_= 11.6, *P* < 10^−4^; aquatic, S1: F_1, 101_= 10.5, *P* = 0.001; terrestrial, S1: F_1, 100_ = 43.5, *P* < 10^−4^), and was higher near the experimental pools (aquatic, S2: F_1, 101_ = 14.7, *P* < 10^−4^; aquatic, S1: F_1, 101_ = 16.2, *P* < 10^−4^; terrestrial, S1: F_1, 100_ = 2.9, *P* = 0.08). All three dependencies were weaker in terrestrial chironomids. Main qualitative differences between the flight patterns of both groups occurred in their responses to the collinear air temperature and relative humidity (Fig. 3 and Additional file 4: Figure S7). While the final models showed that flight activity of aquatic taxa decreased significantly with relative humidity (model S2: F_1, 101_ = 4.8, *P* = 0.02) or tended to increase with temperature (model S1: F_1, 101_ = 3.0, *P *= 0.08), terrestrial taxa had a unimodal response to air temperature with a maximum around 18 °C (model S1: F_2, 100_ = 6.3, *P* = 0.003). Cloud cover did not have a significant effect on either group in models that received substantial support. All three final models again explained large proportions of variability in the data (Table 1).

**Fig. 4:**
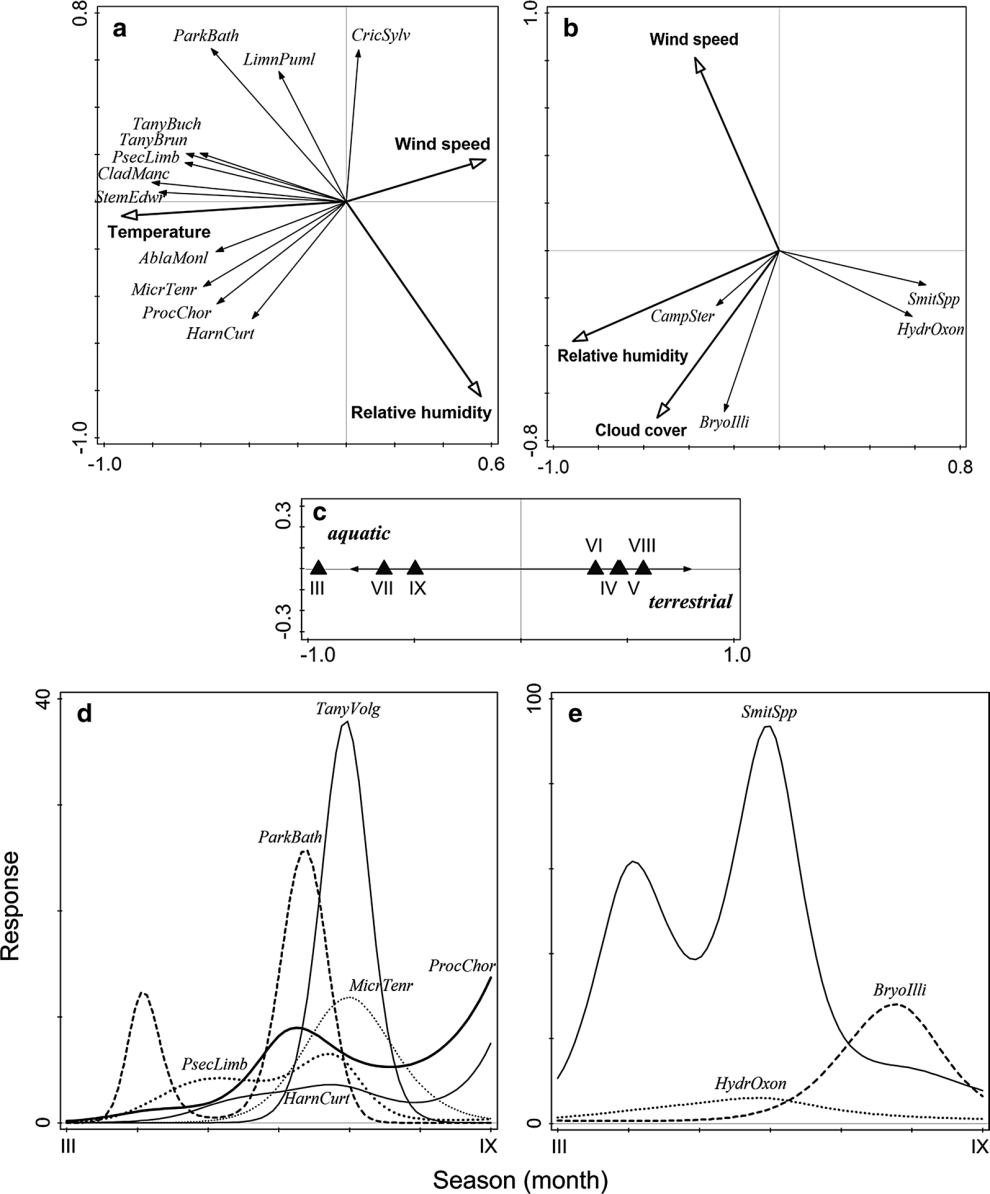
Seasonal flight patterns of chironomids: **a**, **b** response to environmental parameters of **a** aquatic species (RDA, axis 1 = 30.2%, axis 2 = 14.5% of total explained variance), and **b** terrestrial species (RDA, axis 1 = 25.4%, axis 2 = 21.3% of total explained variance), **c** differences between aquatic and terrestrial species (RDA, axis 1 = 63.9% of total explained variance), **d**, **e** species response curves for taxa with more than 60 individuals for **d** aquatic species (RDA, axis 1 = 40.0%, axis 2 = 17.2% of total explained variance), and **e** terrestrial species (RDA, axis 1 = 42.8%, axis 2 = 22.4% of total explained variance). Degrees of freedom (see “Methods” for details): *HarnCurt* = 4, *MicrTenr* = 3, *ParkBath* = 5, *ProcChor* = 5, *PsecLimb* = 5, *TanyVolg* = 3, *BryoIlli* = 3, *HydrOxon* = 3, *SmitSpp* = 5. See Additional file 3: Table S5 for species abbreviations

Community composition of both aquatic and terrestrial assemblage also changed markedly between months and varied with environmental conditions (Table 2 and Fig. 4). Most aquatic species flew mainly under conditions characterized by light wind, higher temperature and low humidity (Fig. 4a). Composition of the terrestrial assemblage changed with humidity, wind speed and cloud cover. Unlike the aquatic species, some common terrestrial species flew preferably in higher humidity conditions (Fig. 4b). When analysed together, the flight patterns of aquatic and terrestrial taxa differed only in their seasonality with alternating main periods of emergence of both groups (Fig. 4c; RDA: 53.0% of adjusted variance, pseudo-F = 5.9, *P* = 0.002).

**Table 2:** Summary of multivariate analyses (RDA and partial RDA) of seasonal and weather-dependent changes in the composition of male chironomid assemblages in 2014–2015

Model	Aquatic species			Terrestrial species		
	AEV	Pseudo-F	*P*	AEV	Pseudo-F	*P*
Month	67.3%	9.9	*0.0001*	57.1%	6.3	*0.0001*
Humidity (*H*)	16.0%	6.0	*0.0005*	20.7%	7.3	*0.0006*
Temperature (*T*)	23.1%	8.8	*0.0001*	2.0%	1.5	0.23
Wind speed (*W*)	7.6%	3.2	*0.012*	12.7%	4.5	*0.009*
Cloud cover (*C*)	3.1%	1.8	0.10	13.4%	4.7	*0.007*
Air pressure	0.9%	1.2	0.26	0.1%	1.0	0.38
*T* + TH regime	35.8%	8.3	*0.0001*	–	–	–
*T* + *H*	35.7%	8.2	*0.0001*	–	–	–
*H* + TH regime	33.2%	7.5	*0.0001*	20.7%	4.1	*0.0002*
*T* + *H* + *W*^a^	38.8%	6.5	*0.0001*	–	–	–
*T* + *H* + *W* (month)	0%	1.0	0.55	–	–	–
*H* + *W* + *C*^b^	–	–	–	37.3%	5.8	*0.0002*
*H* + *W* + *C* (month)	–	–	–	18.7%	2.4	*0.038*

Only species with at least five occurrences included. Significant results (*P* < 0.05) in italics. Covariates used in partial RDA given in parentheses. *AEV* adjusted explained variation of the model, *TH regime* one of the two temperature-humidity regimes; see “Methods” for details

^a, b^Shown in Fig. 4

Seasonal flight activity patterns of the most common species (Fig. 4d, e; aquatic species: RDA: 67.9% of adjusted variance, pseudo-*F* = 10.2, *P* < 10^−4^; terrestrial: RDA: 57.1% of adjusted variance, pseudo-F = 6.3, *P* < 10^−4^) were characterized by a more or less narrow, single peak of emergence [aquatic *Tanytarsus volgensis* Miseiko and *Microchironomus tener* (Kieffer), and terrestrial *Bryophaenocladius* cf. *illimbatus* (Edwards)], while other taxa had more protracted periods of flight activity [aquatic: *Psectrocladius* gr. *limbatellus*; terrestrial: *Hydrosmittia oxoniana* (Edwards)] or were indicative of two generations per year [aquatic: *Parakiefferiella bathophila* (Kieffer), *Harnischia curtilamellata* (Malloch), and *Procladius choreus* (Meigen)].

Earlier studies often linked changes in flight activity and species composition to temperature or other weather conditions alone. We thus used constrained partial ordination test to detect the conditional effects of the significant environmental variables (*P* < 0.05) after accounting for the seasonality as a covariate to obtain comparable results. Surprisingly, we found no conditional effect of weather conditions on the species composition of aquatic species, which contrasted with a significant conditional effect of humidity, wind speed and cloud cover on the composition of terrestrial species (Table 2). Moreover, variation partitioning confirmed a strong effect of seasonality that dominated over the joint effect of temperature, humidity, wind speed and air pressure for both aquatic (total explained variation: season only, 13.2%; weather only, 2.9%; shared, 4.9%) and terrestrial species (total explained variation: season only, 24.6%; weather only, 10.0%; shared, 24.0%).

## Discussion

Our study provides a detailed quantitative analysis of flight activity patterns of adult chironomids in the temperate zone. We found that the patterns vary strongly in time and with weather conditions. Previous studies of adult chironomids investigated the impact of landscape heterogeneity on their spatial distribution [34, 35] or aimed to identify key environmental drivers of their flight activity [24]. However, earlier data analyses, such as Pearson correlation or linear regression [24], did not explicitly consider seasonality that reflects larval phenology. Moreover, we directly compared the activity of aquatic and terrestrial species of the family, which can indicate if larval habitat modulates the drivers of adult flight activity. Any seasonal differences between the groups should be driven by the timing of emergence and reflect both constraints on life cycles and habitat-specific differences in thermal regimes, food availability, and other factors that affect individual ontogenies.

Little is known on the detailed motion of flying adult chironomids [27]. Although we could not determine if the individuals performed a short local flight, possibly to join a mating swarm or to find resting or feeding places, or a directional flight from further away [24], females were 2.3 times as abundant as males in our data. Males typically dominate in mating swarms [29] and we are thus confident that our data reflect dispersal or the feeding phase preceding dispersal [27] rather than just swarming activity. In other words, the observed flight patterns in our study, especially those of females, were likely driven by incoming or outgoing individuals that were dispersing from the natal habitat and sought new breeding sites. Moreover, we found significantly more individuals of aquatic but not of terrestrial species near the experimental pools. This agrees with the spatial dilution effect of aquatic chironomids (i.e., their abundance should decline with distance from water when individuals fly in multiple directions [35]) as opposed to the terrestrial species, for which conditions at both sites were comparable.

### Temporal flight activity patterns: daily and seasonal scales

Flight patterns of both aquatic and terrestrial chironomids in our study depended strongly on daytime and season, even after accounting for weather conditions. Chironomids at our study site flew preferably during the evening and afternoon, with another lower maximum in the morning. Previous studies on chironomids also reported that the timing of adult emergence closely coincides with species-specific flight activity of adults, and broadly classified them into day- and night-flyer groups [16]. Daytime group flies mainly around noon and afternoon, whereas the activity of crepuscular species often peaks during evening and after dark [49], with dusk as the preferred period for oviposition flight of females [27]. Our results resemble the diel patterns of biting and flight activity of mosquitoes [50] but contrast with those of aquatic beetles and bugs, which fly predominantly in mid-morning, at noon, and at dusk [5, 7]. Our results thus support the assumption that flight in the evening under higher relative humidity reduces the risk of dehydration [5, 50], which should be particularly important for chironomids (but see below), even if this timing may compromise the possibility of long-distance dispersal by air turbulences and convection that occur more often during the day [16]. Other factors such as high predation risk by insectivorous birds during the day may have also contributed to the lack of a mid-day peak in flight activity observed in our study.

We found that seasonality is the main driver of long-term changes in overall flight activity and species composition of adult chironomids, although weather conditions also have a substantial impact as in previous studies (e.g., [12, 23]). Moreover, we observed an alternating seasonal pattern between terrestrial and aquatic species in both 2013 and 2014–2015 datasets. The pattern in 2013 could have been partly driven by the early successional stage and a limited local species pool, but we observed a similar outcome in 2014–2015, with the aquatic species more common in March, July and September and terrestrial species more common in the other months. We hypothesize that this alternating pattern directly reflects larval phenology and the role of environmental constraints such as seasonal differences in food availability [51], thermal conditions, and predation pressure [27] in the aquatic and terrestrial ecosystems.

Dominance of seasonality over weather conditions was particularly obvious in the species composition of aquatic species. Differences in the taxonomic resolution of the data or more depauperate terrestrial community might underlie this result, because rapid turnover of many taxa in the aquatic community could have favoured seasonality as the main explanatory variable. On the other hand, we have probably underestimated turnover in the terrestrial community for which we could only achieve lower taxonomic resolution.

Adult chironomid lifespan is very short, often less than one day and rarely up to 1–2 weeks [29]. Their flight activity is thus mostly determined by the timing of emergence [52]. High-resolution data on phenology requires long-term use of emergence traps [29], which we could not deploy at our freely accessible site. However, the flight patterns observed in our study indicate that we did not miss important events, at least not for the commonest species. Our data are consistent with a univoltine life cycle and more or less narrow summer emergence period in *T.* *volgensis* and *M. tener*, bivoltine life cycle with two emergence periods in *H. curtilamellata* (summer and autumn) and especially in *P. bathophila* (spring and summer), and protracted emergence with increased abundance in summer in *P.* gr. *limbatellus*. These patterns including peak emergence periods are consistent with data from other regions [53, 54], although they may not hold across the whole distribution area (e.g., *P.* *bathophila* was reported to have up to 3–4 generations in Bavarian lakes; [55]).

### Dependence of flight activity patterns on weather conditions

Contrary to expectations, weather conditions affected diel and seasonal flight activity patterns in aquatic and terrestrial chironomids differently. Total flight activity of both groups changed with air temperature and humidity, but the responses were not identical. Terrestrial taxa consistently flew most at intermediate temperatures and relative humidity levels, which likely reflects the opposing pressures of air temperature and humidity on flight activity outside a certain optimal range. This unimodal response to temperature in terrestrial taxa contrasts with a positive temperature-biomass relationship in Arctic chironomids [24] that is likely driven by the much lower temperatures. Together, these results suggest a unimodal relationship between temperature and flight activity of chironomids across a wide range of temperatures.

We identified no air temperature and humidity optima for aquatic taxa. Their total flight activity declined with relative humidity on both diel and seasonal timescales. Although similar result were found for aquatic beetles and heteropteran bugs [18], previous studies on dipterans found purely or predominantly positive relationships between relative humidity and flight activity [17, 21, 22]. Aquatic taxa in our study also flew less at higher temperatures at the diel timescale, which contrasted with an increasing trend in flight activity at higher temperatures across the season. Humidity but not temperature affected the species composition of terrestrial community and neither had an effect on the aquatic community. Previous studies reported that aquatic insects fly more at higher air temperatures (Trichoptera: [11]; Heteroptera: [56]; Plecoptera: [12]; Chironomidae: [24]) or lower humidity (Coleoptera and Heteroptera: [18]), although the responses were sometimes sex (*Culicoides*: [22]) or species specific (*Culicoides*: [23]) or varied in time (*Simulium*: [21]). Seasonality of air temperature is also responsible for seasonal changes in diel activity patterns in Coleoptera and Heteroptera [6]. However, earlier analyses ignored the collinearity of weather characteristics (but see [12, 18]) and their seasonal variation (but see [12, 23]).

Rising wind speeds strongly inhibited flight activity of adult chironomids despite a few species flying in stronger winds [> ca. 3.5 m s^−1^; e.g., *Cricotopus sylvestris* (F.)]. The mechanism that allows those species to fly in such conditions could not be identified because we could not distinguish if the individuals in the samples were only attempting to take off (in which case they would likely stop flying if the wind speeds exceeded their airspeed) or if they were able to use brief lulls to take off and were already flying. Estimated effects of wind speed on diel activity patterns differed in both aquatic and terrestrial taxa. On the daily timescale, both groups were most abundant under light wind conditions that presumably facilitate dispersal over larger distances [27], and their flight activity declined above wind speeds of ca. 2.5 m s^−1^ and stopped above 3 m s^−1^ similar to biting midges [23]. On the seasonal timescale, total flight activity of both groups also declined with increasing wind speeds. This is consistent with reported strong declines in flight activity with rising wind speeds or a complete cessation of flight above a certain wind threshold in stoneflies [12], water beetles and bugs [13, 14, 18, 57], biting midges [22], black flies [21], and swarming chironomids [58]. In our data, the decline was steeper and wind speed explained a substantial proportion of variability in species composition in terrestrial species.

Finally, more individuals of both aquatic and terrestrial taxa flew under higher air pressure, which characterizes good and stable weather. This pattern was observable only on the seasonal timescale, i.e., could be partly due to a correlation of air pressure with seasonal temperature, and was stronger in aquatic species. Cloud cover affected species composition of terrestrial species but had no measurable effect on total flight activity of either group as in biting midges [23], mayflies and caddisflies [31]. This could have resulted from the collinearity between cloud cover and relative humidity (Additional file 2: Table S3) that could mask the role of cloud cover. Interestingly, no effect of air pressure or cloud cover was found on the flight activity of Arctic chironomids [24].

## Conclusions

Overall, our results imply that species phenologies and conditions experienced by the larvae dominate adult flight patterns of chironomids and probably also other short-lived aquatic insects. This may underpin the evolution of diversification of life history strategies such as cohort splitting in which at least some adults should experience favourable conditions [32]. Long-term studies coupling detailed observations of local environmental conditions both in and out of water, larval dynamics and adult emergence and flight activity at a given site would be thus particularly useful to disentangle the effects of different biotic and abiotic drivers on the life histories and population dynamics of aquatic chironomids and other aquatic insect groups. Our study also reiterates that individual weather variables are often strongly collinear and their effects on flight activity are difficult to separate. Teasing apart the importance of collinear environmental variables would benefit from the use of controlled experiments (as in [17]), but these may not be feasible for all taxa. We recommend using a more comprehensive set of statistical models in future studies of environmental drivers of insect dispersal that would cover the joint effect of air temperature and humidity and go beyond simple linear relationships.

## Additional files

**Additional file 1.** Map of the study area and additional information on the sampling protocols (**Figures S1** and **S2** and **Table S1**).

**Additional file 2.** Weather conditions (**Figures S3** and **S4** and **Tables S2**–**S4**).

**Additional file 3.** Adult chironomids collected during the study (**Table S5**).

**Additional file 4.** Additional results (**Figures S5**–**S7**).
