# Supplementary material for: Seasonality and weather conditions jointly drive flight activity patterns of aquatic and terrestrial chironomids
Source: BMC Ecol. 2018 Jun 19;18:19. doi: 10.1186/s12898-018-0175-y (PMC6006739; doi:10.1186/s12898-018-0175-y)
Supplement: Supplementary file 1 — Additional file 1. Map of the study area and additional information on the sampling protocols (Figures S1 and S2 and Table S1). [file 12898_2018_175_MOESM1_ESM.docx]

**Additional file 1: Map of the study area and additional information on the sampling protocols (Figures S1 and S2 and Table S1)**.

**Figure S1** Schematic map of the study area showing experimental pools (grey circles), lake (grey area), vegetation (dotted area), steep slopes (checkerboard), drains (parallel lines), position of Malaise traps (x), and sweeping route (dashed line) within locations (A and B) in the sandpit


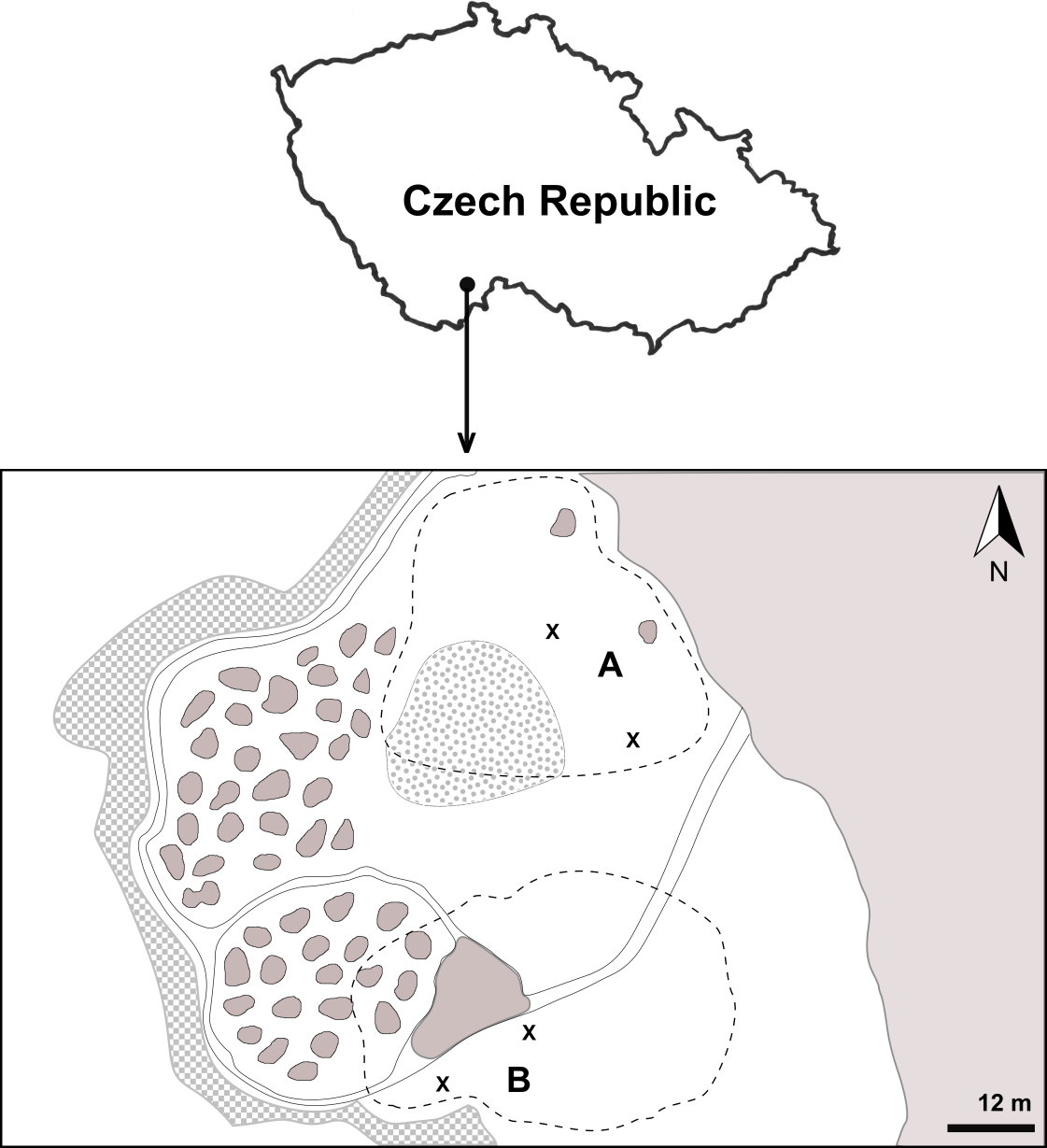


**Figure S2** Malaise trap used in the study. The trap (effective intercept area = 8.7 m^2^) consisted of the main trapezoid vertical panel (black fine mesh, heights 1.85 m and 1 m, upper edge 2.07 m long, lower edge 1.80 m long) bordered by two larger (1.06 x 0.68 m) and two smaller (0.92 x 0.6 m) perpendicular panels (black fine mesh) at each side of the panel and a sloping roof (white fine mesh). Experimental pools and the steep slope shown in Fig. 1 are visible in the background


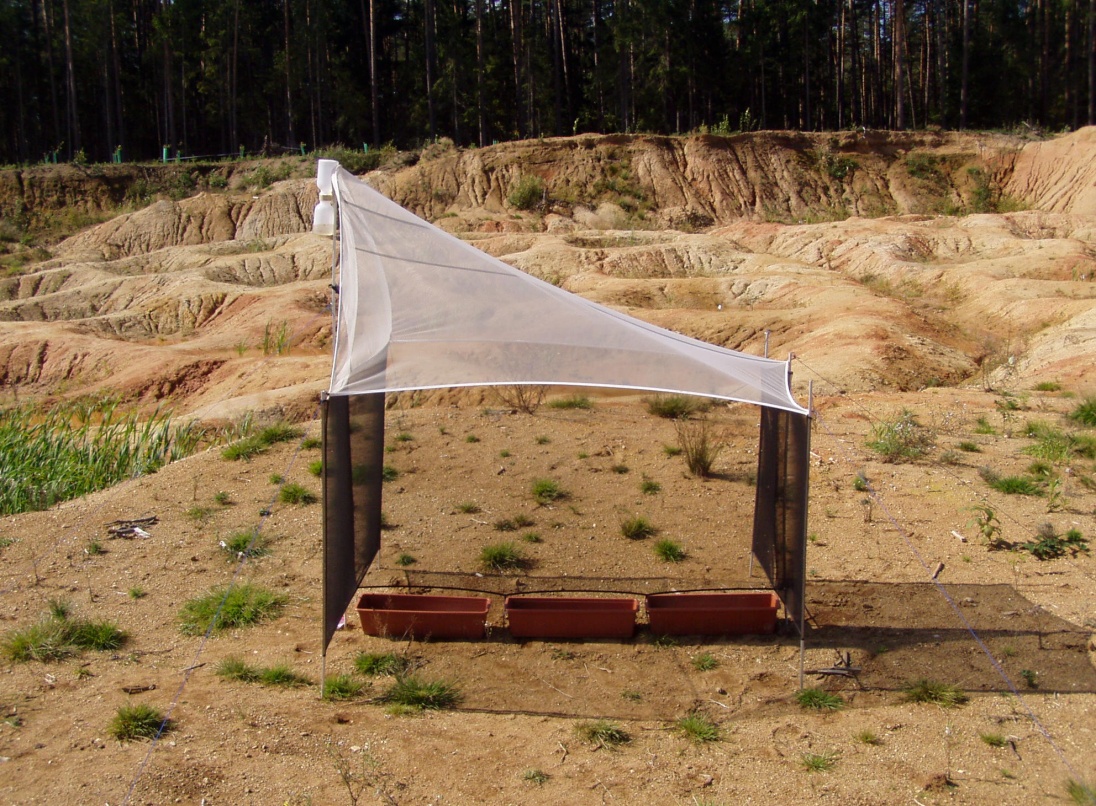


**Table S1** Overview of spatiotemporal resolution and pooling of samples in the analyses of the 2013 and 2014–2015 datasets. See Methods for details

| **Dataset** | **Method** | **Temporal resolution** | **Spatial resolution** |
| --- | --- | --- | --- |
| Handnet 2013 | univariate analyses | 1 hour (11 per day) | separate routes  (1 per each hour) |
| Handnet vs. Malaise traps 2013 | rarefaction analyses | pooled daily data | (a) all sites across each method pooled  (b) separate sites for both methods |
| Malaise traps 2014–2015 | univariate analyses | pooled afternoon and evening data (1 per day) | separate traps  (4 per day) |
|  | multivariate analyses, species response curves, trait-based response analysis | pooled afternoon and evening data (1 per day) | all traps pooled  (1 per day) |
