# Supplementary material for: Seasonality and weather conditions jointly drive flight activity patterns of aquatic and terrestrial chironomids
Source: BMC Ecol. 2018 Jun 19;18:19. doi: 10.1186/s12898-018-0175-y (PMC6006739; doi:10.1186/s12898-018-0175-y)
Supplement: Supplementary file 2 — Additional file 2. Weather conditions (Figures S3 and S4 and Tables S2–S4). [file 12898_2018_175_MOESM2_ESM.docx]

**Additional file 2: Weather conditions (Figures S3 and S4 and Tables S2–S4)**

**Figure S3** Air temperature and relative humidity recorded in 2013 at the study site in the sandpit during each 15-minute handnet sampling interval in August (circles) and September (triangles). Data points = average values for the 15-minute intervals, colour coded by hours

**
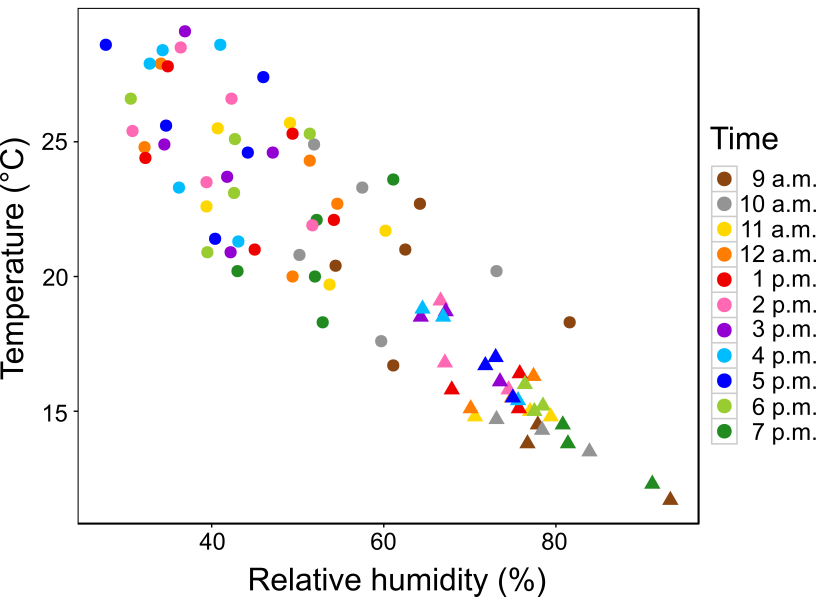
**

**Table S2** Weather conditions during the sampling dates in 2013 and 2014–2015. Summary based on point data measured on the site once during each 15-minute sampling interval for the 2013 dataset and on average values for the sampling interval (14:30 to 22:30) for the 2014–2015 dataset. Data for wind speed, cloud cover, and air pressure for 2014–2015 were provided by the Czech Hydrometeorological Institute weather station in Třeboň. Cloud cover data from 2013 and 2014–2015 are on different scales; categories for 2013 data: 1 = clear sky, 2 = mostly sunny, 3 = mostly cloudy, and 4 = cloudy (see Methods for details)

| **Variable** | **2013** | | |  | **2014**–**2015** | | |
| --- | --- | --- | --- | --- | --- | --- | --- |
|  | **Mean ± SD** | **Min** | **Max** |  | **Mean ± SD** | **Min** | **Max** |
| Temperature (°C) | 19.9 ± 4.4 | 9.5 | 30.5 |  | 17.4 ± 8.1 | 5.2 | 29.8 |
| Relative humidity (%) | 58.8 ± 17.7 | 25.8 | 97.3 |  | 59.3 ± 18.3 | 34.0 | 80.8 |
| Wind speed (m.s^-1^) | 2.9 ± 1.1 | 0.1 | 3.8 |  | 2.4 ± 1.4 | 0.1 | 5.9 |
| Cloud cover | 2.9 ± 0.98 | 1 | 4 |  | 5 ± 3.2 | 0 | 9 |
| Air pressure (hPa) | - | - | - |  | 970.8 ± 4.5 | 962.9 | 979.1 |

**Table S3** Correlation of average weather conditions (Avg) with their respective minima (Min) and maxima (Max) during the sampling intervals in the 2014–2015 data. See Methods for details. Significant results (*P* < 0.05) in bold; *r* = correlation coefficient; CI = 95% confident interval; *n* = 28

|  |  | **Min** ~ **Avg** | **Max** ~ **Avg** |
| --- | --- | --- | --- |
| **Temperature** | ***r*** | 0.94 | 0.98 |
|  | **CI** | (0.87; 0.97) | (0.96; 0.99) |
|  | ***P*** | **< 10^-4^** | **< 10^-4^** |
|  |  |  |  |
| **Relative humidity** | ***r*** | 0.95 | 0.78 |
|  | **CI** | (0.88; 0.97) | (0.57; 0.89) |
|  | ***P*** | **< 10^-4^** | **< 10^-4^** |
|  |  |  |  |
| **Wind speed** | ***r*** | 0.93 | 0.91 |
|  | **CI** | (0.85; 0.97) | (0.81; 0.96) |
|  | ***P*** | **< 10^-4^** | **< 10^-4^** |
|  |  |  |  |
| **Cloud cover** | ***r*** | 0.95 | 0.94 |
|  | **CI** | (0.89; 0.98) | (0.88; 0.97) |
|  | ***P*** | **< 10^-4^** | **< 10^-4^** |
|  |  |  |  |
| **Air pressure** | ***r*** | 1.00 | 0.86 |
|  | **CI** | (0.99; 1.00) | (0.72; 0.93) |
|  | ***P*** | **< 10^-4^** | **< 10^-4^** |

**Figure S4** Relationship between values recorded in 2014–2015 at the study site in the sandpit (*y*) and at the meteorological station in the town of Třeboň (*x*) for (a) temperature: *y* = 1.09 *x* + 0.37 (*r^2^* = 0.78), (b) relative humidity: *y* = 0.62 *x* + 19.31 (*r^2^* = 0.63), (c) wind speed: *y* = 1.13 *x* + 1.95 (*r^2^* = 0.71), and (d) two temperature-humidity regimes detected in sandpit with linear regression of temperature *T* plotted against relative humidity *H*: *T* = -0.32 *H* + 40.8 (*r^2^* = 0.92) in May–September 2014 (upper band) and *T* = -0.31 *H* + 27.3 (*r^2^* = 0.89) in March and April 2015 (lower band). Data points = average values for the daily sampling intervals (14:30–22:30); dashed lines = regression lines; colour coded by months


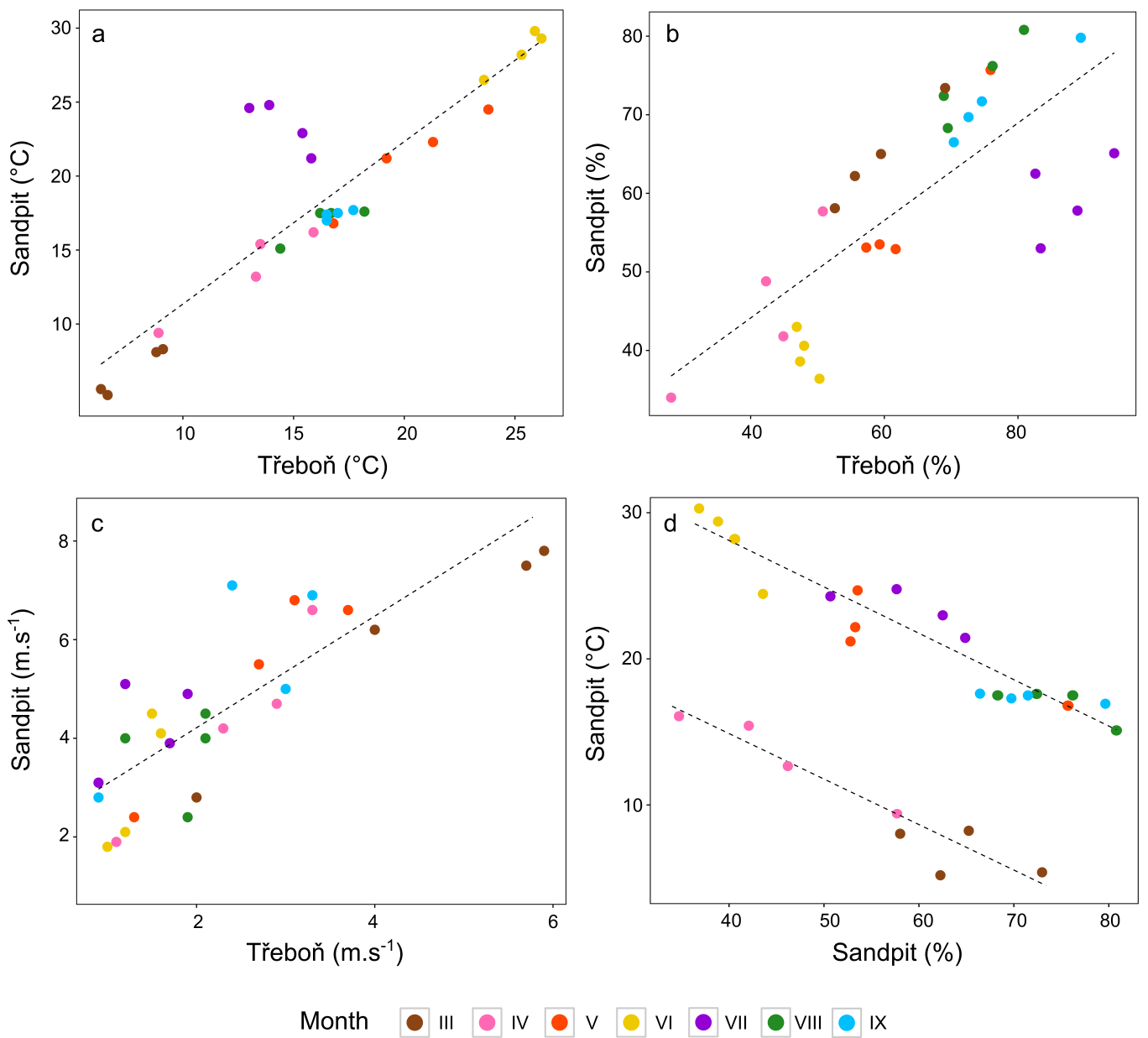


**Table S4** Pearson correlation coefficients of weather variables used in the analyses of the 2014–2015 data. Significant results (*P* < 0.05) in bold; *r* = correlation coefficient; CI = 95% confidence interval; *n* = 28. Correlation between air temperature and relative humidity pools the two weather regimes together; see Methods for details

|  |  | **Temperature** | **Relative humidity** | **Wind speed** | **Cloud cover** |
| --- | --- | --- | --- | --- | --- |
| **Relative humidity** | ***r*** | -0.46 |  |  |  |
|  | **CI** | (-0.71; -0.10) |  |  |  |
|  | ***P*** | **0.013** |  |  |  |
|  |  |  |  |  |  |
| **Wind speed** | ***r*** | -0.54 | 0.15 |  |  |
|  | **CI** | (-0.76; -0.20) | (-0.24; 0.49) |  |  |
|  | ***P*** | **0.003** | 0.45 |  |  |
|  |  |  |  |  |  |
| **Cloud cover** | ***r*** | -0.29 | 0.69 | 0.21 |  |
|  | **CI** | (-0.60; 0.09) | (0.43; 0.85) | (-0.18; 0.54) |  |
|  | ***P*** | 0.13 | **< 10^-4^** | 0.28 |  |
|  |  |  |  |  |  |
| **Air pressure** | ***r*** | -0.46 | -0.28 | 0.43 | -0.12 |
|  | **CI** | (-0.71; -0.11) | (-0.59; 0.11) | (0.07; 0.69) | (-0.47; 0.27) |
|  | ***P*** | **0.013** | 0.15 | **0.022** | 0.55 |
