# Supplementary material for: Seasonality and weather conditions jointly drive flight activity patterns of aquatic and terrestrial chironomids
Source: BMC Ecol. 2018 Jun 19;18:19. doi: 10.1186/s12898-018-0175-y (PMC6006739; doi:10.1186/s12898-018-0175-y)
Supplement: Supplementary file 3 — Additional file 3. Adult chironomids collected during the study (Table S5). [file 12898_2018_175_MOESM3_ESM.docx]

**Additional file 3: Adult chironomids collected during the study (Table S5)**

**Table S5** Total abundances and habitat associations of chironomid taxa captured by the two methods in 2013 and in 2014–2015

| **Taxon** | **Habitat** | **Species code** | **Handnet 2013** | **Malaise traps 2013** | **Malaise traps 2014–15** |
| --- | --- | --- | --- | --- | --- |
| **Chironominae** |  |  |  |  |  |
| *Chironomus* sp. | (aqua) |  | 0 | 0 | 4 |
| *Chironomus obtusidens* Goetghebuer, 1921 | aqua |  | 0 | 1 | 6 |
| *Chironomus plumosus* (Linnaeus, 1758) | aqua |  | 0 | 0 | 5 |
| *Chironomus riparius* agg. Meigen, 1804 | aqua |  | 0 | 0 | 4 |
| *Chironomus* cf. *venustus* Pinder, 1978 | aqua |  | 0 | 0 | 4 |
| *Cladopelma edwardsi* (Kruseman, 1933) | aqua |  | 0 | 1 | 2 |
| *Cladopelma virescens* (Kruseman, 1933) | aqua |  | 0 | 1 | 1 |
| *Cladotanytarsus* sp. | (aqua) |  | 0 | 0 | 5 |
| *Cladotanytarsus atridorsum* Kieffer, 1924 | aqua |  | 0 | 0 | 1 |
| *Cladotanytarsus bicornutus* Kieffer, 1922 | aqua |  | 0 | 0 | 1 |
| *Cladotanytarsus mancus* (Walker, 1856) | aqua | *CladManc* | 21 | 96 | 38 |
| *Cladotanytarsus* cf. *molestus* Hirvenoja, 1962 | aqua |  | 0 | 0 | 1 |
| *Cladotanytarsus vanderwulpi* (Edwards, 1929) | aqua |  | 0 | 0 | 2 |
| *Cryptotendipes* cf. *usmaensis* (Pagast, 1931) | aqua |  | 1 | 2 | 7 |
| *Demicryptochironomus vulneratus* (Zetterstedt, 1838) | aqua |  | 0 | 0 | 2 |
| *Dicrotendipes pulsus* (Walker, 1856) | aqua |  | 0 | 1 | 1 |
| *Endochironomus tendens* (Fabricius, 1775) | aqua |  | 0 | 1 | 1 |
| *Glyptotendipes* sp. | (aqua) |  | 0 | 0 | 1 |
| *Glyptotendipes pallens* (Meigen, 1804) | aqua |  | 0 | 1 | 1 |
| *Glyptotendipes* cf. *paripes* (Edwards, 1929) | aqua |  | 0 | 0 | 2 |
| *Glyptotendipes signatus* (Kieffer, 1909) | aqua |  | 0 | 0 | 1 |
| *Harnischia curtilamellata* (Malloch, 1915) | aqua | *HarnCurt* | 2 | 17 | 70 |
| *Microchironomus tener* (Kieffer, 1918) | aqua | *MicrTenr* | 5 | 15 | 78 |
| *Micropsectra atrofasciata* (Kieffer, 1911) | aqua |  | 0 | 0 | 2 |
| *Micropsectra lindrothi* Goetghebuer, 1931 | aqua |  | 0 | 0 | 2 |
| *Microtendipes pedellus* (De Geer, 1776) | aqua |  | 0 | 0 | 6 |
| *Paracladopelma laminatum* (Kieffer, 1921) | aqua |  | 0 | 0 | 4 |
| *Parachironomus* sp. | (aqua) |  | 0 | 1 | 1 |
| *Polypedilum* sp. 1 | (aqua) |  | 0 | 1 | 0 |
| *Polypedilum* sp. 2 | (aqua) |  | 0 | 0 | 1 |
| *Polypedilum bicrenatum* Kieffer, 1921 | aqua |  | 0 | 1 | 1 |
| *Polypedilum nubeculosum* (Meigen, 1804) | aqua |  | 0 | 0 | 1 |
| *Polypedilum nubifer* (Skuse, 1889) | aqua |  | 792 | 45 | 0 |
| *Polypedilum sordens* (van der Wulp, 1875) | aqua |  | 0 | 0 | 1 |
| *Polypedilum tritum* (Walker, 1856) | aqua |  | 0 | 0 | 4 |
| *Polypedilum* *uncinatum* (Goetghebuer, 1921) | aqua |  | 0 | 0 | 1 |
| *Rheotanytarsus muscicola* (Thienemann, 1929) | aqua |  | 0 | 1 | 2 |
| *Saetheria reissi* Jackson, 1977 | aqua |  | 0 | 0 | 1 |
| *Stempellinella edwardsi* Spies & Saether, 2004 | aqua | *StemEdwr* | 2 | 2 | 29 |
| *Stictochironomus* sp. | (aqua) |  | 0 | 3 | 10 |
| Tanytarsini gen. sp. | (aqua) |  | 0 | 0 | 2 |
| *Tanytarsus* sp. | (aqua) |  | 0 | 0 | 2 |
| *Tanytarsus bathophilus* Kieffer, 1911 | aqua |  | 0 | 1 | 12 |
| *Tanytarsus brundini/curticornis* | aqua | *TanyBrun* | 0 | 0 | 9 |
| *Tanytarsus buchonius* Reiss & Fittkau, 1971 | aqua | *TanyBuch* | 0 | 0 | 31 |
| *Tanytarsus chinyensis* Goetghebuer, 1934 | aqua |  | 0 | 0 | 10 |
| *Tanytarsus eminulus* (Walker, 1856) | aqua |  | 0 | 0 | 2 |
| *Tanytarsus lestagei* agg. (Lindeberg, 1967) | aqua |  | 0 | 0 | 2 |
| *Tanytarsus lugens/gregarius* | aqua |  | 0 | 0 | 1 |
| *Tanytarsus mendax* Kieffer, 1925 | aqua |  | 0 | 0 | 2 |
| *Tanytarsus pallidicornis* (Walker, 1856) | aqua |  | 0 | 0 | 4 |
| *Tanytarsus signatus* (van der Wulp, 1859) | aqua |  | 0 | 0 | 1 |
| *Tanytarsus striatulus* Lindeberg, 1976 | aqua |  | 0 | 1 | 0 |
| *Tanytarsus sylvaticus* (van der Wulp, 1859) | aqua |  | 0 | 0 | 8 |
| *Tanytarsus volgensis* Miseiko, 1967 | aqua | *TanyVolg* | 11 | 95 | 160 |
| *Virgatanytarsus* sp. | (aqua) |  | 0 | 1 | 2 |
| **Tanypodinae** |  |  |  |  |  |
| *Ablabesmyia* sp. | (aqua) |  | 0 | 0 | 2 |
| *Ablabesmyia longistyla* Fittkau, 1962 | aqua |  | 2 | 3 | 11 |
| *Ablabesmyia monilis* (Linnaeus, 1758) | aqua | *AblaMonl* | 1 | 1 | 21 |
| *Conchapelopia melanops* (Meigen, 1818) | aqua |  | 0 | 0 | 1 |
| *Conchapelopia viator* (Kieffer, 1911) | aqua |  | 0 | 0 | 4 |
| *Macropelopia adaucta* Kieffer, 1916 | aqua |  | 0 | 0 | 4 |
| *Procladius* sp. | (aqua) |  | 0 | 0 | 1 |
| *Procladius choreus* (Meigen, 1804) | aqua | *ProcChor* | 2 | 81 | 148 |
| *Procladius* cf. *flavifrons* Edwards, 1929 | aqua |  | 0 | 0 | 2 |
| *Procladius sagittalis* (Kieffer, 1909) | aqua |  | 0 | 1 | 4 |
| **Orthocladiinae** |  |  |  |  |  |
| *Acricotopus lucens* (Zetterstedt, 1850) | aqua |  | 0 | 6 | 0 |
| *Allocladius bothnicus* (Tuiskunen, 1984) | terr |  | 0 | 0 | 3 |
| *Allosmittia* sp. | (terr) |  | 4 | 1 | 4 |
| *Bryophaenocladius ictericus* (Meigen, 1830) | terr |  | - | - | 4 |
| *Bryophaenocladius* cf. *illimbatus* (Edwards, 1929) | terr | *BryoIlli* | 1 | 3 | 217 |
| *Bryophaenocladius* nr. *simus* (Edwards, 1929) | terr |  | - | - | 4 |
| *Bryophaenocladius nitidicollis* (Goetghebuer, 1913) | terr |  | - | - | 2 |
| *Camptocladius stercorarius* (De Geer, 1776) | terr | *CampSter* | 0 | 1 | 17 |
| *Cricotopus* sp. | (aqua) |  | 0 | 0 | 1 |
| *Cricotopus* cf. *albiforceps* (Kieffer, 1916) | aqua |  | 0 | 0 | 1 |
| *Cricotopus brevipalpis* Kieffer, 1909 | aqua |  | 3 | 8 | 2 |
| *Cricotopus festivellus* (Kieffer, 1906) | aqua |  | 0 | 0 | 5 |
| *Cricotopus intersectus* (Staeger, 1839) | aqua |  | 0 | 5 | 7 |
| *Cricotopus sylvestris* (Fabricius, 1794) | aqua | *CricSylv* | 0 | 2 | 26 |
| *Cricotopus vierrensis* Goetghebuer, 1935 | aqua |  | 2 | 2 | 0 |
| *Eukiefferiella* sp. | (aqua) |  | 0 | 0 | 1 |
| *Heterotrissocladius marcidus* (Walker, 1856) | aqua |  | 0 | 0 | 1 |
| *Hydrosmittia oxoniana* (Edwards, 1922) | terr | *HydrOxon* | 3 | 0 | 79 |
| *Limnophyes* sp. | (aqua) |  | 2 | 6 | 17 |
| *Limnophyes asquamatus* Andersen, 1937 | ND |  | 0 | 1 | 0 |
| *Limnophyes pumilio* (Holmgren, 1869) | aqua | *LimnPuml* | 2 | 6 | 23 |
| *Metriocnemus* 4 spp. | ND |  | 0 | 1 | 20 |
| *Orthocladius* sp. | (aqua) |  | 0 | 0 | 2 |
| *Parakiefferiella bathophila* (Kieffer, 1912) | aqua | *ParkBath* | 44 | 31 | 118 |
| *Paraphaenocladius impensus* (Walker, 1856) | semi |  | 1 | 2 | 0 |
| *Psectrocladius bisetus* Goetghebuer, 1942 | aqua |  | 0 | 0 | 2 |
| *Psectrocladius* gr. *limbatellus* Wuelker, 1956 † | aqua | *PsecLimb* | 0 | 14 | 64 |
| *Psectrocladius platypus* (Edwards, 1929) | aqua |  | 0 | 0 | 1 |
| *Pseudorthocladius* sp. | (terr) |  | - | - | 3 |
| *Pseudosmittia* sensu lato | (terr) |  | 3 | 0 | 1 |
| *Pseudosmittia holsata* Thienemann & Strenzke, 1940 | terr |  | - | - | 4 |
| *Pseudosmittia mathildae* Albu, 1968 | ND |  | - | - | 2 |
| *Rheocricotopus* sp. | (aqua) |  | 0 | 0 | 2 |
| *Rheosmittia spinicornis* (Brundin, 1956) | aqua |  | 0 | 0 | 1 |
| *Smittia* spp. ‡ | (terr) | *SmitSpp* | - | - | 954 |
| *Tvetenia* sp. | (aqua) |  | 0 | 0 | 18 |
| unidentified terrestrial Orthocladiinae § | terr |  | 675 | 432 | - |
|  |  |  |  |  |  |
| Total number of aquatic males |  |  | 890 | 449 | 1042 |
| Total number of semi- and terrestrial males |  |  | 687 | 439 | 1292 |
| Ratio aquatic males : total males (%) |  |  | 56.4 | 50.4 | 44.2 |
| Total number of females ¶ |  |  | - | - | 5286 |

† *P. limbatellus* (Holmgren, 1869), *P. oxyura* Langton, 1985, and *P. oligosetus* Wuelker, 1956 are present at the site.

‡ Mostly *S. edwardsi* Goetghebuer, 1932, but *S. aterrima* (Meigen, 1818) and *S. leucopogon* (Meigen, 1804) were also present.

§ Mostly *Smittia* with *Bryophaenocladius* and one species of *Hydrosmittia*.

¶ Females were not considered in the analyses.

Abbreviations: *aqua* = aquatic larvae, semi = semi-terrestrial larvae living mostly outside or near water but requiring high humidity, *terr* = terrestrial larvae, *ND* = no data on larval habitat type; habitat data in parentheses = dominant habitat preference of other members of the genus. Habitat preferences based on [1–4]. Some terrestrial Orthocladiinae taxa were not distinguished in the identification and no females were counted in the 2013 dataset; these data are entered as ‘-’. *Chironomus* sp., *Cladotanytarsus* sp., *Glyptotendipes* sp., *Tanytarsus* sp., *Ablabesmyia*sp., *Procladius* sp., *Cricotopus*sp., and *Limnophyes* sp. refer to unidentifiable specimens that most likely belonged to one of the other recorded species from the respective genus.

## References

1. Fittkau EJ, Reiss F. Chironomidae. In: Illies J, editor. Limnofauna Europaea. A checklist of the animals inhabiting European inland waters, with accounts of their distribution and ecology (except Protozoa), 2nd edn. Stuttgart: Gustav Fisher Verlag; 1978. p. 404–440.

2. Moller Pillot, HKM. Chironomidae larvae of the Netherlands and adjacent lowlands: biology and ecology of the Chironomini. Zeist, Netherlands: KNNV Publishing; 2009.

3. Moller Pillot, HKM. Chironomidae larvae of the Netherlands and adjacent lowlands: biology and ecology of the aquatic Orthocladiinae. Zeist, Netherlands: KNNV Publishing; 2013.

4. Vallenduuk HJ, Moller Pillot HKM. Chironomidae larvae of the Netherlands and adjacent lowlands: general ecology and Tanypodinae. Zeist, Netherlands: KNNV Publishing; 2013.
