# Supplementary material for: Seasonality and weather conditions jointly drive flight activity patterns of aquatic and terrestrial chironomids
Source: BMC Ecol. 2018 Jun 19;18:19. doi: 10.1186/s12898-018-0175-y (PMC6006739; doi:10.1186/s12898-018-0175-y)

**Additional file 4: Supplementary results (Figures S5–S7)**

**Figure S5** Rarefaction curves (data: solid lines, extrapolation: dashed lines) of the handnet (blue and violet lines), Malaise trap (red and green lines) for (a) whole chironomid assemblage and (b) only for aquatic species with data disaggregated between the sites near the lake shore (locality A, red and blue lines) and close to the southern edge of the experimental pools (locality B, green and violet lines). Shaded areas indicate 95 % confidence intervals

**
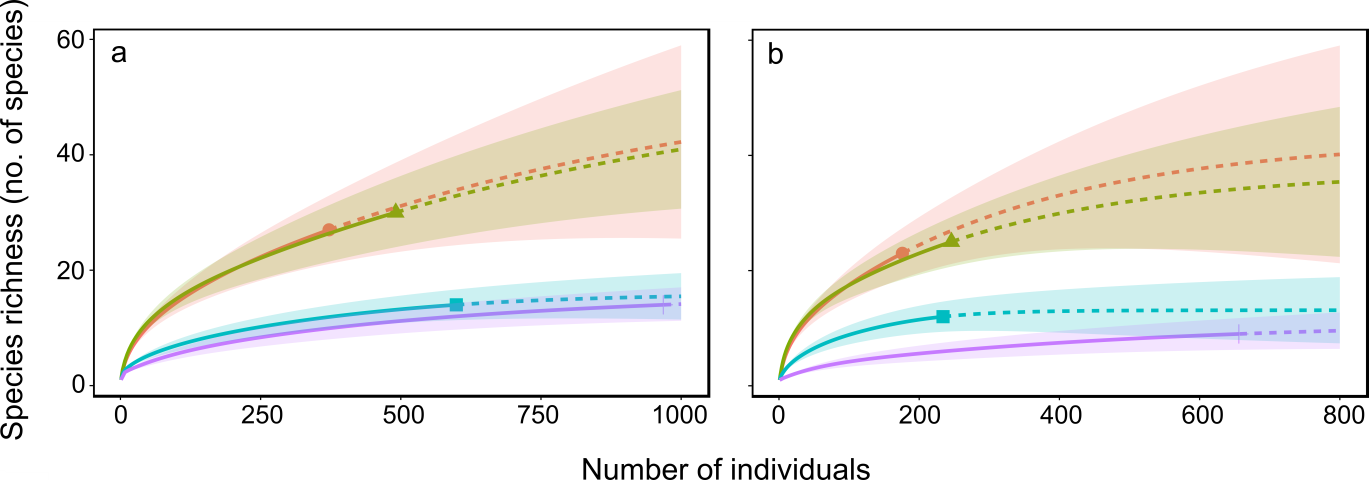
**

**Figure S6** Drivers of diel patterns of total flight activity of (a–d) aquatic and (e–g) terrestrial species according to the second best model (D2). Symbols and axes as in Fig. 2


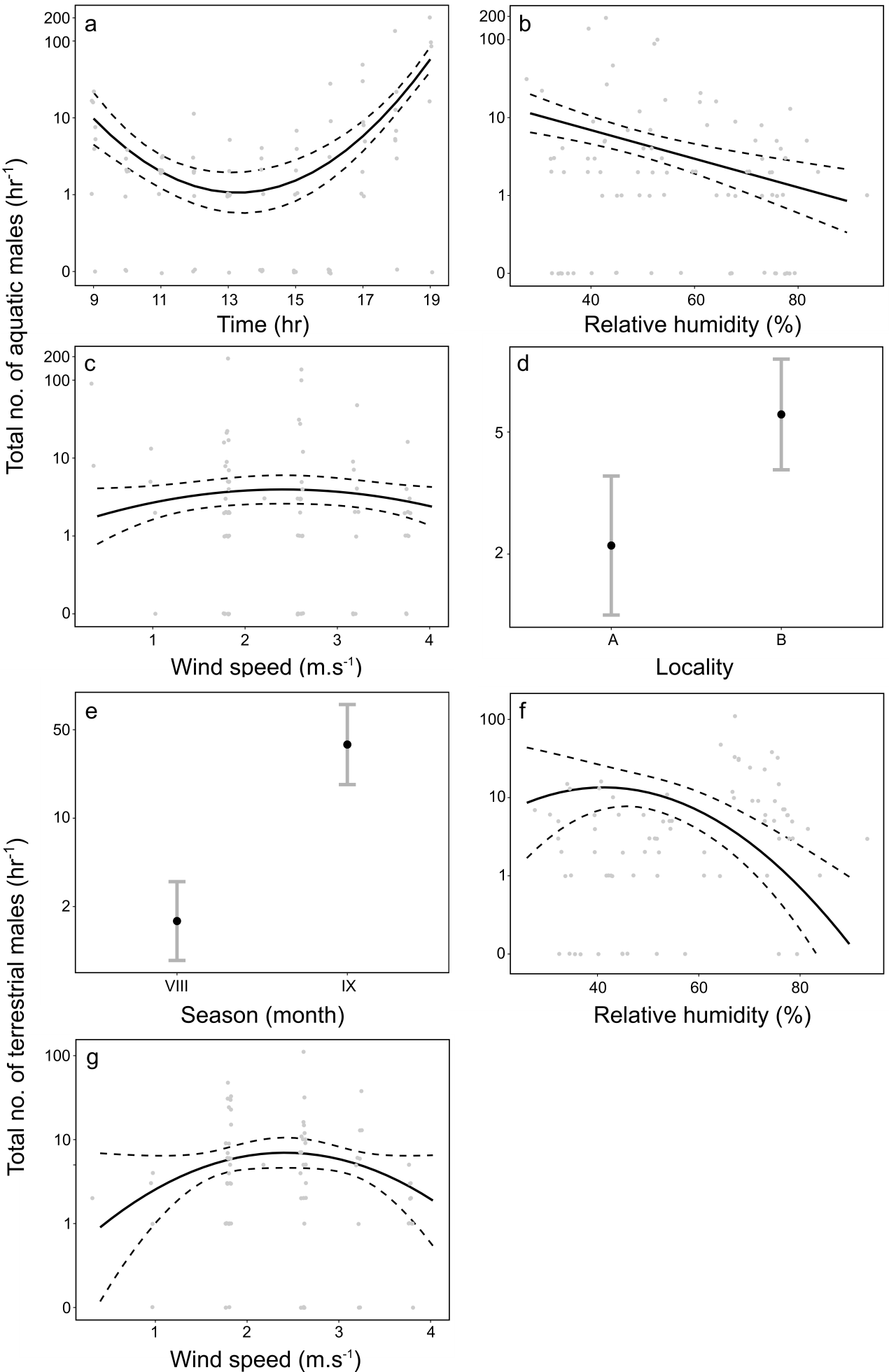


**Figure S7** Drivers of seasonal patterns of total flight activity of aquatic species (a–e) according to the second best model (S1). Symbols and axes as in Fig. 2


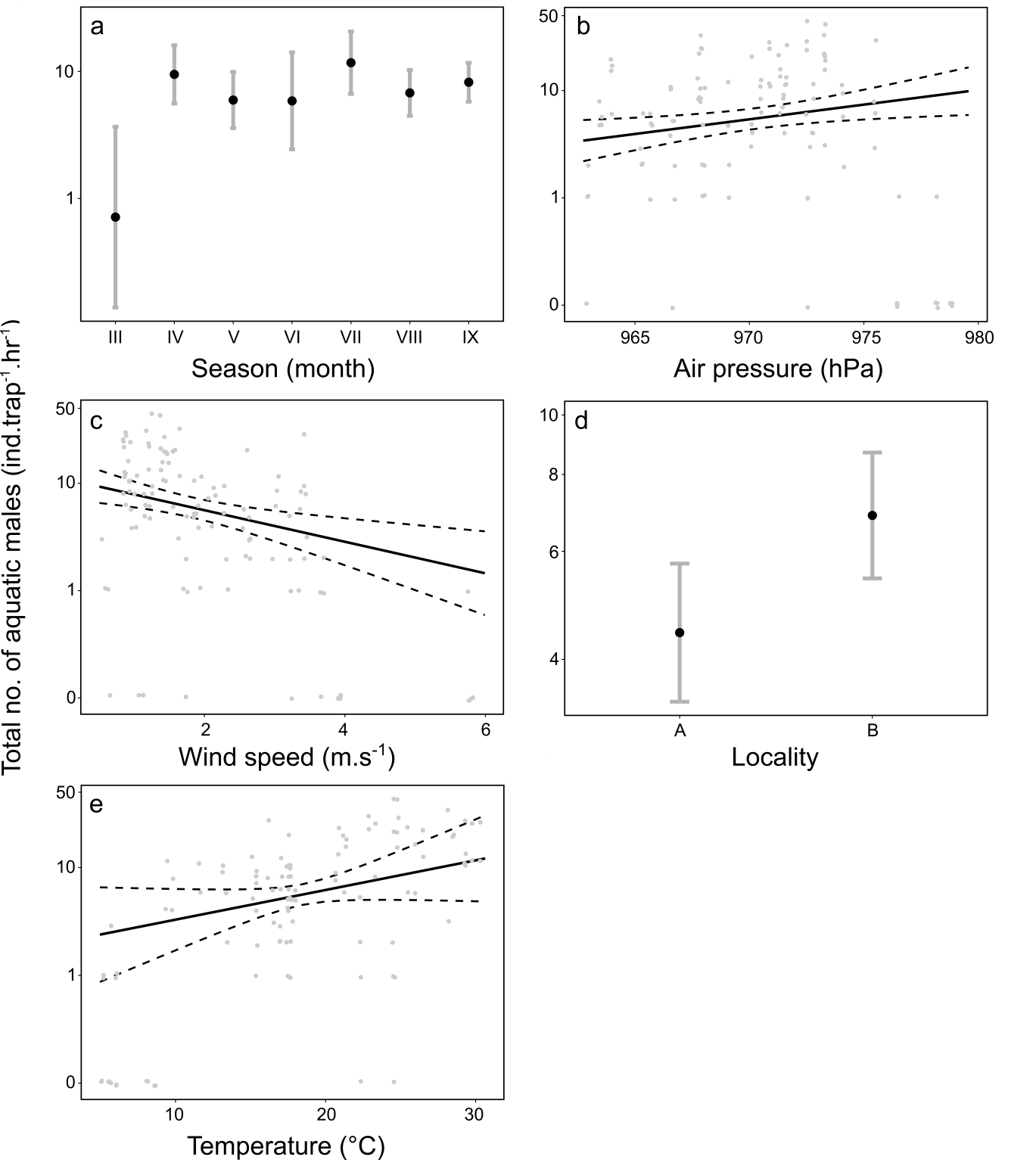

Supplement: Supplementary file 4 — Additional file 4. Additional results (Figures S5–S7). [file 12898_2018_175_MOESM4_ESM.docx]
